# Porifera collection of the Italian National Antarctic Museum (MNA), with an updated checklist from Terra Nova Bay (Ross Sea)

**DOI:** 10.3897/zookeys.758.23485

**Published:** 2018-05-15

**Authors:** Claudio Ghiglione, Maria Chiara Alvaro, Matteo Cecchetto, Simonepietro Canese, Rachel Downey, Alice Guzzi, Claudio Mazzoli, Paola Piazza, Hans Tore Rapp, Antonio Sarà, Stefano Schiaparelli

**Affiliations:** 1 Italian National Antarctic Museum (MNA), Section of Genoa, Italy Italian National Antarctic Museum Genoa Italy; 2 Department of Earth, Environmental and Life Science (DISTAV), University of Genoa, Italy University of Genoa Genoa Italy; 3 Institute for Environmental Protection and Research, ISPRA, Milazzo, Italy Institute for Environmental Protection and Research Milazzo Italy; 4 Australia National University, Fenner School of Environment and Society, Canberra, Australia Australia National University Canberra Australia; 5 Department of Geosciences, University of Padova, Italy University of Padova Padova Italy; 6 Department of Physical, Earth and Environmental Sciences, University of Siena, Italy University of Siena Siena Italy; 7 Department of Biological Sciences and K.G. Jebsen Centre for Deep-Sea Research, University of Bergen, Norway University of Bergen Bergen Norway; 8 Studio Associato Gaia S.N.C., Via Brigata Liguria, Genoa, Italy Studio Associato Gaia S.N.C. Genoa Italy

**Keywords:** Antarctica, Italian National Antarctic Museum, Porifera, Ross Sea, Terra Nova Bay, 3D models

## Abstract

This new dataset presents occurrence data for Porifera collected in the Ross Sea, mainly in the Terra Nova Bay area, and curated at the Italian National Antarctic Museum (MNA, section of Genoa). Specimens were collected in 331 different sampling stations at depths ranging from 17 to 1,100 meters in the framework of 17 different Italian Antarctic expeditions funded by the Italian National Antarctic Research Program (PNRA). A total of 807 specimens, belonging to 144 morphospecies (i.e., 95 taxa identified at species level and 49 classified at least at the genus level) is included in the dataset. Nearly half (45%) of the species reported here correspond to species already known for Terra Nova Bay. Out of the remaining 55% previously unknown records, under a third (~29%) were classified at the species level, while over a quarter (~26%) were ascribed to the genus level only and these would require further study. All vouchers are permanently curated at the MNA and are available for study to the scientific community. A 3D model of an uncommon species from the Ross Sea, i.e. *Tethyopsisbrondstedi* (Burton, 1929), is also presented and will be made available for outreach purposes.

## Purpose

Since the very beginning of the Italian expeditions in the Ross Sea, which started in 1985, sponges have been one of the most studied taxa, due both to the high number of species found in the Terra Nova Bay area (where Italy has its coastal station “Mario Zucchelli”, 74°41’42”S, 164°7’23”E), and to the strong community of Italian taxonomists specialized in Antarctic sponges. This sponge collection has been progressively growing each year, with new collections of specimens at each expedition of the Italian National Antarctic Research Program (PNRA) in the Terra Nova Bay area. These specimens have been studied and exchanged among different researchers for comparisons and publications for years, they represented the base of a PhD thesis (Sarà 2002), and then the whole collection was finally acquired by the Italian National Antarctic Museum (MNA, section of Genoa, Italy) in 2010. Since then the sponge collection has been restored, crosschecked for distributional data with the original labels, matched with a collection of permanent glass slides of spicules and updated in terms of taxonomy or new identifications of specimens.

This study aims at publishing and valorising occurrence data of the Porifera collected during several scientific expeditions of the Italian National Antarctic Program (PNRA) in the Ross Sea.

This collection is amongst the largest for Antarctic sponges and despite it being mainly focused on the Terra Nova Bay area, it represents a unicum given the amount of permanent glass slides with spicules available and the large database of images of sponges documented *in situ*, or freshly collected.

This distributional dataset is the fifth MNA contribution to the Antarctic Biodiversity Portal (www.biodiversity.aq), which is the thematic Antarctic node for both the Ocean Biogeographic Information System (AntOBIS) and the Global Biodiversity Information Facility (ANTABIF), based on materials stored at the MNA. The previous contributions were: Ghiglione et al. (2013), Piazza et al. (2014), Selbmann et al. (2015) and Cecchetto et al. (2017).

## Project description

**Project title**: Antarctic Porifera in the collection of the Italian National Antarctic Museum (MNA)

**Curator and Promoter**: Stefano Schiaparelli

**Personnel**: Claudio Ghiglione, Maria Chiara Alvaro, Matteo Cecchetto, Simonepietro Canese, Rachel Downey, Alice Guzzi, Claudio Mazzoli, Paola Piazza, Hans Tore Rapp, Antonio Sarà, Stefano Schiaparelli

**Funding**: The specimens were collected during different Antarctic expeditions funded by the Italian National Antarctic Research Program (PNRA). The complete list of research projects is reported here (in italic is the project name or category under the PNRA program, followed by the project code, the expedition number, and the corresponding year):

• *Necton e risorse da pesca* 2.1.4.6, III expedition (1987/1988)

• *Oceanografia & Benthos* 2.1.4.3, III expedition (1987/1988)

• *Benthos* 3.2.1.2.5, V expedition (1989/1990)

• *Oceanografia geologica* 3.2.1.4, V expedition (1989/1990)

• *Ecologia e biogeochimica dell’Oceano Meridionale* 2d.2, IX expedition (1993/1994)

• *Ecologia e biogeochimica dell’Oceano Meridionale* 2d.2, X expedition (1994/1995)

• *Ecologia e biogeochimica dell’Oceano Meridionale – ROSSMIZE* 2d.2, XI expedition (1995/1996)

• *Ecologia e biogeochimica dell’Oceano Meridionale* 2b.3, XIII expedition (1997/1998)

• *Struttura e dinamica delle cenosi marine di Baia Terra Nova* 2b.3.1, XIV expedition (1998/1999)

• *L’area marina protetta di Baia Terra Nova: struttura e variazioni a breve e lungo termine* 8.5, XV expedition (1999/2000)

• *Processi genetici e significato paleoclimatico e paleoceanografico dei CARBONati marini biogenici in ANTartide – CARBONANT* 4.7, XVII expedition (2001/2002)

• *L’area marina protetta di Baia Terra Nova: struttura e variazioni a breve e lungo termine* 8.5, XVII expedition (2001/2002)

• *The costal ecosystem of Victoria Land coast: distribution and structure along the latitudinal gradient* 2002/8.6, XVIII expedition (2002/2003)

• *The costal ecosystem of Victoria Land coast: distribution and structure along the latitudinal gradient* 2002/8.6, XIX expedition (2003/2004)

• *Batteri e cianobatteri antartici: biodiversità e produzione di composti con potenzialità applicative in biotecnologia* 2004/1.6, XX expedition (2004/2005)

• *Variabilità della ventilazione polare abissale e suo impatto sulla circolazione globale – PolarDOVE* 2004/8.2, XXI expedition (2005/2006)

• *L’ecosistema costiero di Baia Terra Nova – Latitudinal Gradient Project* 2006/08.01, XXV expedition (2009/2010)

• *Ecologia e ciclo vitale di specie ittiche costiere del Mare di Ross* 2004/08.04, XXV expedition (2009/2010)

• *Barcoding of Antarctic Marine Biodiversity – BAMBi* 2010/A1.10, XXVII expedition (2011/2012)

• *Diversità genetica spazio temporale di endoparassiti delle regioni polari: uno studio per la valutazione dell’impatto dei cambiamenti globali sulle reti trofiche marine* 2009/A1.09, XXVIII expedition (2012/2013)

• *Barcoding of Antarctic Marine Biodiversity – BAMBi* 2010/A1.10, XXVIII expedition (2012/2013)

• *Vulnerabilità dei pesci polari al cambiamento climatico: ciclo vitale*, *habitats e relazione con il ghiaccio marino in Pleuragramma antarcticum* 2010/A1.11, XXVIII expedition (2012/2013)

• *Barcoding of Antarctic Marine Biodiversity – BAMBi* 2010/A1.10, XXIX expedition (2013/2014)

• *Integrità dell’ecosistema marino antartico come presupposto per lo studio dell’interazione parassita-ospite: un approccio genetico*, *molecolare ed immunologico* 2013/AZ1.09, XXIX expedition (2013/2014)

**Figure 1.**
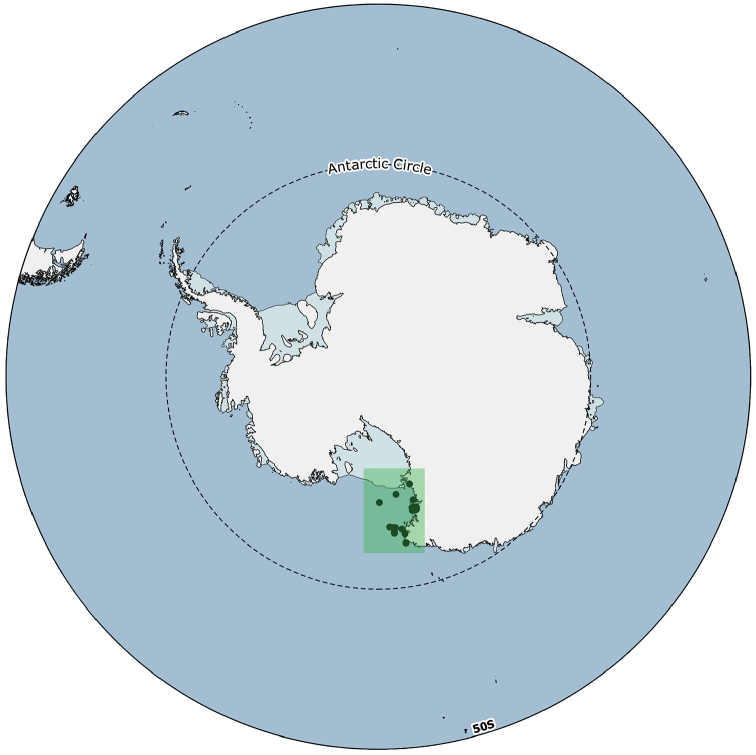
General map of Antarctica with the study area highlighted (green box). Detailed maps of the sampling areas are provided in Figs 3, 4, and 5.

**Study area description**: The specimens were collected in the Ross Sea sector of the Southern Ocean in a bathymetric range from 17 to 1,100 meters of depth (Fig. 1).

**Design description**: Data were assembled by revising all the distributional records of the specimens deposited in the collections of the Italian Antarctic National Museum (MNA, section of Genoa, Italy). The samples were collected in the framework of several expeditions of the Italian National Antarctic Research Program (PNRA) from 1987 to 2014.

## Methods

**Figure 2.**
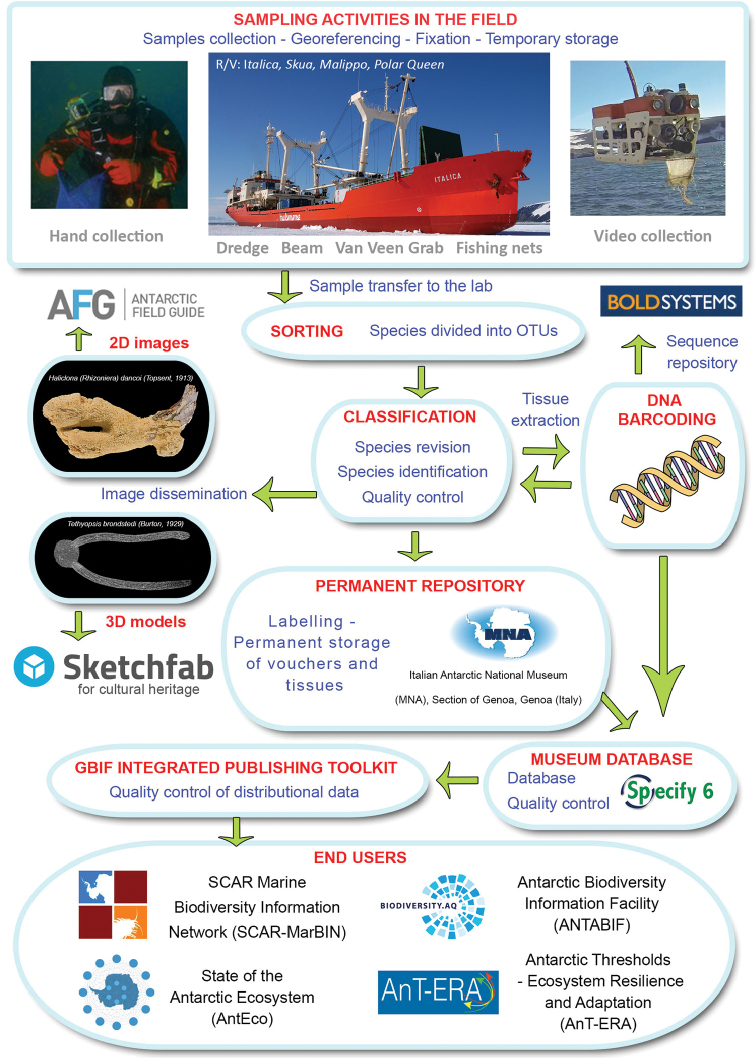
Flowchart depicting major stages in dataset development and publishing.

**Method step description**: See sampling description below and flowchart of Fig. 2.

**Figure 3.**
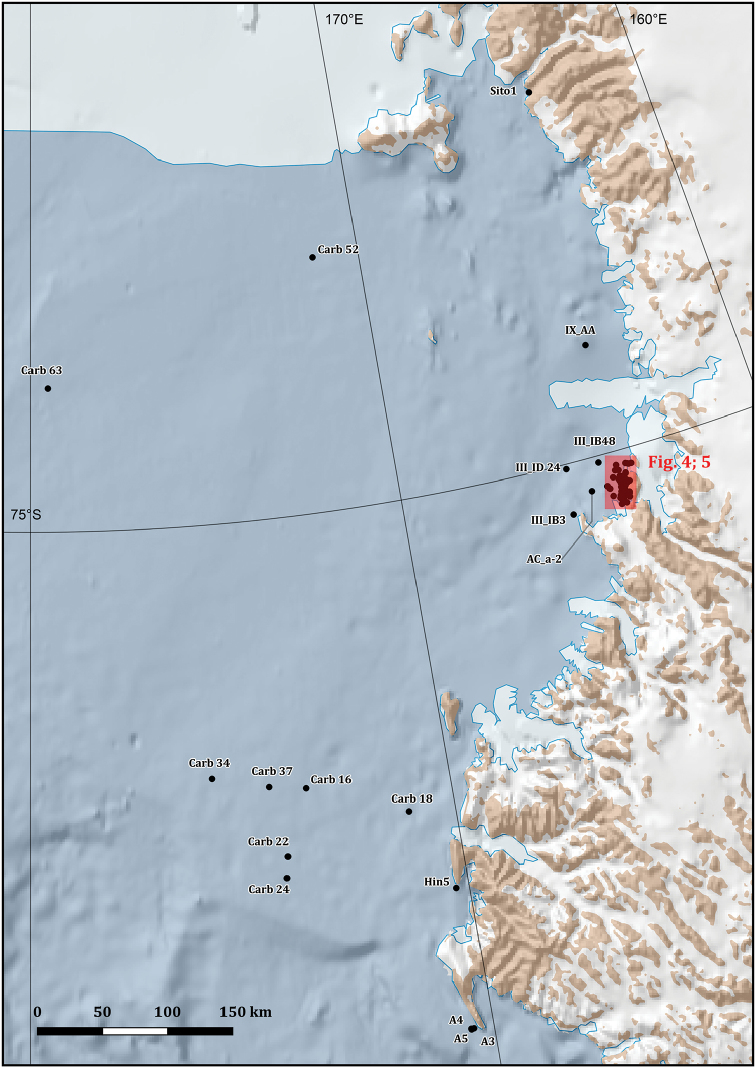
Sampling stations map in the Ross Sea area, Antarctica. The area in the red box is depicted, at a finer spatial scale, in Figs 3, 4.

**Figure 4.**
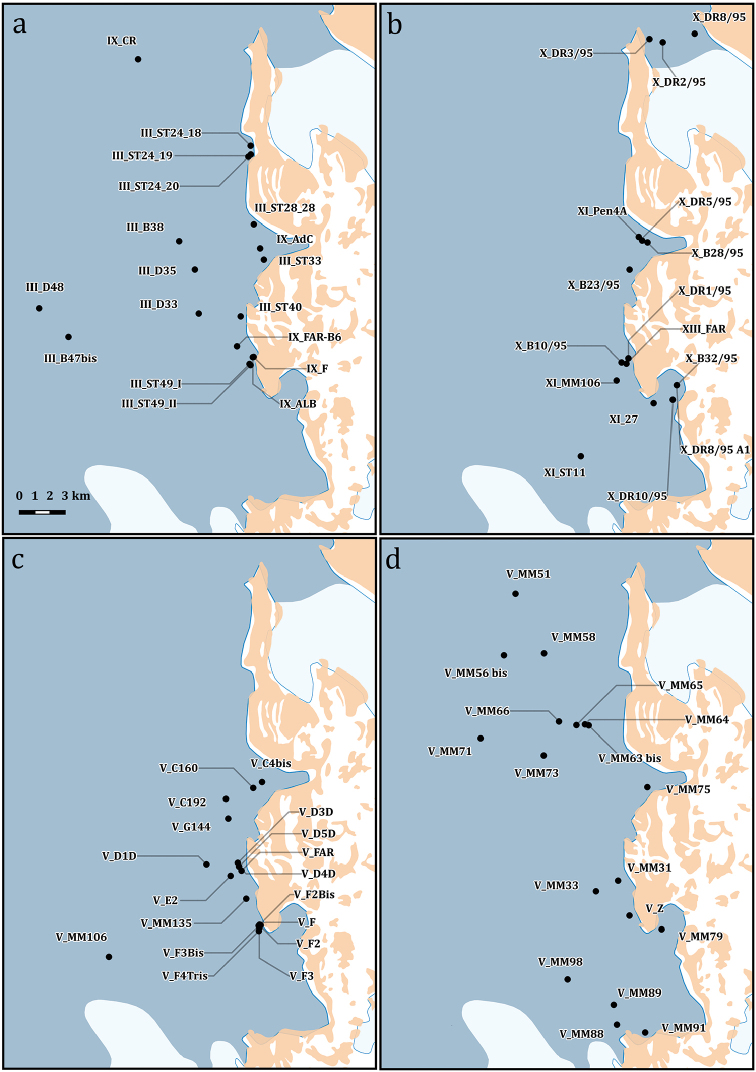
Detailed map of the sampling stations of the PNRA expeditions III, V, IX, X, XI, XIII.

**Figure 5.**
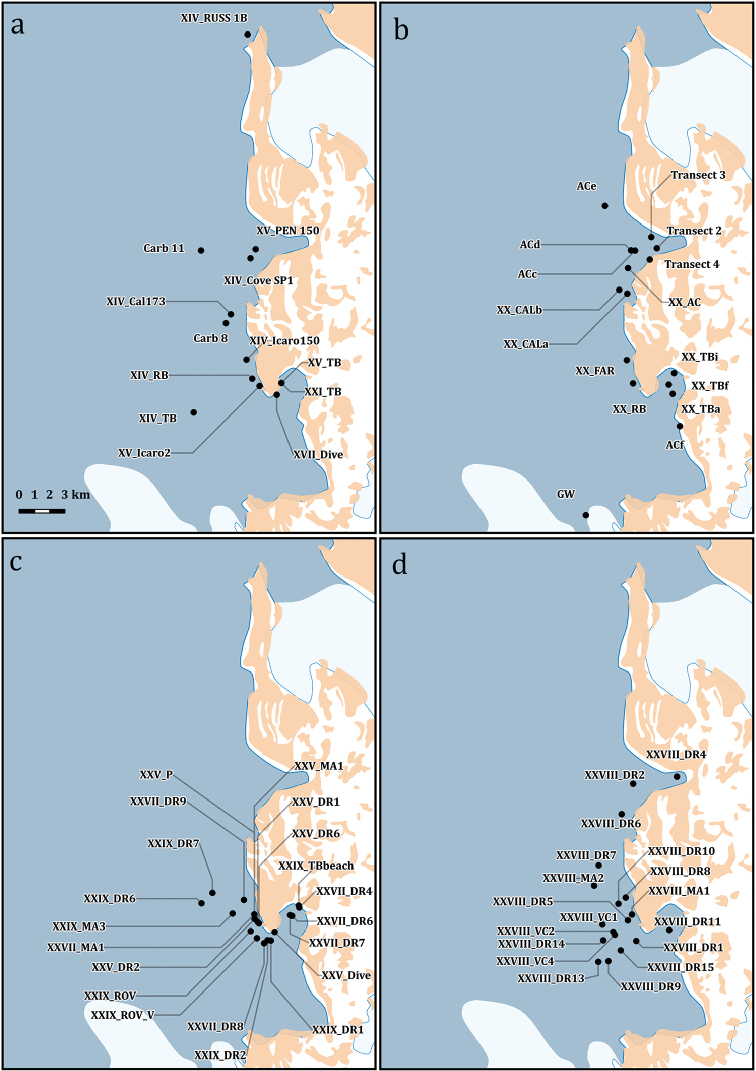
Detailed map of the sampling stations of the PNRA expeditions II XIV, XV, XVII, XX, XXI, XXV, XXVII, XXVIII, XXIX.

**Study extent description**: The distributional data considered here originated from 331 different sampling stations ranging between 17 and 1,100 metres of depth (Figs 1, 3, 4, 5).

**Figure 6.**
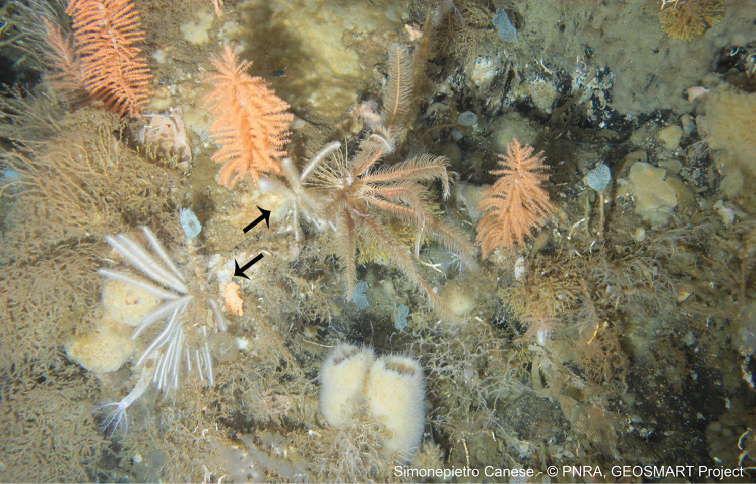
ROV video frame reporting the presence (arrows) of the species Lycopodinacf.vaceleti (van Soest & Baker, 2011) at Terra Nova Bay.

**Sampling description**: Sampling was performed on a total of 331 different sampling stations (Figs 1, 3, 4, 5) through the deployment of a variety of sampling gears, mainly dredges (Charcot dredge, Naturalist dredge, Triangular dredge and Picard dredge) and Van Veen grabs of different volumes. Some samples were also opportunistically collected by long fishing lines, mid water trawls (that touched the bottom due to a failure of the winches), trammel nets, and other fishing nets that provided additional material to standard techniques. Some samples, from the XIV, XV, XVII, XVIII, XXIII, and XXV PNRA expeditions, were hand-collected by SCUBA diving. In one case (i.e., *Sycettaantarctica* (Brøndsted, 1931), MNA 8847) the specimen was collected beached and sampling coordinates refer to the coastline. In another case, i.e., Lycopodinacf.vaceleti (van Soest and Baker 2011), the specimen record is based on a georeferenced ROV video frame (Fig. 6) and no physical samples are available.

Once the material has been acquired by the MNA after sorting and shipment activities, all the specimens were classified to the lowest possible taxonomical resolution. In the years, different researchers have contributed to the classification of the specimens: Homoscleromorpha were studied by Maurizio Pansini and Antonio Sarà; Demospongiae and Hexactinellida were studied by Rachel Downey, Antonio Sarà, Marco Bertolino, Maurizio Pansini and Barbara Calcinai; Calcarea were studied by Hans Tore Rapp.

The present dataset has been formatted in order to fulfil the Darwin Core standard protocol required by the OBIS scheme (http://www.iobis.org/manual/lifewatchqc/) and according to the SCAR-MarBIN Data Toolkit (available at http://www.scarmarbin.be/documents/SM-FATv1.zip). The dataset was uploaded in the ANTOBIS database (the geospatial component of SCAR-MarBIN).

Vouchers are now preserved in 90% ethanol (~53% of the entire collection), frozen (~23%), or dried (~24%). The data flow chart illustrating the sampling, sorting, and storing procedures for specimens, data, and image availability is reported in Fig. 2.

**Quality control description**: Specimens were identified at the finest possible taxonomic resolution and only those that have been classified at least at the genus level were included in the present dataset. During all the phases of sorting, classification, and storage of samples at the MNA, quality control and data cleaning have been undertaken at various stages in order to produce high quality data and make consistent cross-references between the database and sample labels. The MNA uses an SQL-based database (Specify 6) and a R-Shiny web application to manage its collections and link all the data (photos, glass slides, etc.) to the physical samples.

Due to the large amount of researchers that managed the material before the acquisition of the collection by the MNA, all the specimens and distributional records were rechecked and then imported in the museum database. During this phase it emerged that only ~75% of the Porifera collection fulfilled the expected minimum set of data fields to be included in GBIF. The remaining ~25% of the material present in the MNA collection can be divided in a ~7%, represented by material not yet classified, and another ~18% represented by old materials that cannot be ascribed to a specific sampling station due to missing labels or incomplete information about sampling.

Georeferencing on board each of the different research vessels is based on the interpolation of GPS satellite receivers and a gyrocompass. Station coordinates and sampling events were recorded during sampling activities based on various GPS systems.

## Taxonomic coverage

**Figure 7.**
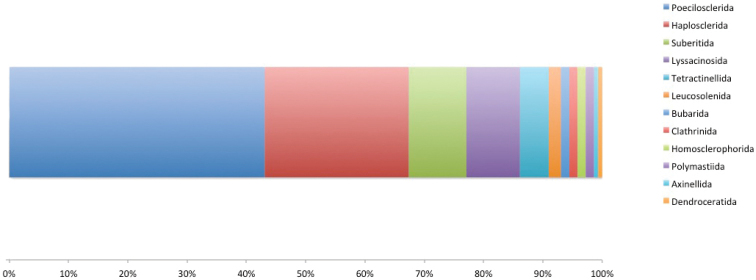
Taxonomic coverage (reported in percentage of specimens per Order) of MNAPorifera collection. Poecilosclerida cover ~43% of the collection specimens, followed by Haplosclerida (~24%), Suberitida (~10%) and Lyssacinosida (~9%). The remaining orders cover less than ~14%.

**General taxonomic coverage description**: This dataset focuses on all classes (Calcarea, Demospongiae, Hexactinellida, and Homoscleromorpha) of the Phylum Porifera (Kingdom Animalia), and includes a total of 807 specimens belonging to 144 morphospecies (with 95 taxa classified at species level and 49 at genus level), and representing 12 orders and 30 families (Fig. 7). Nearly half (~45%; 65 species) of the collected taxa correspond to records already known for the Terra Nova Bay area (Cattaneo et al. 2000; Cerrano et al. 2000; Sarà et al. 1990, 1992), about one third (~29%; 42 species) correspond to new records classified at specific level, and just over a quarter (~26%; 37 species) to new records classified at the genus level. The new records for Terra Nova Bay are reported with the acronym ‘NR’ immediately after the species name in the following taxonomic ranks section.

Permanent glass slides with the spicules are available for ~88% of the species of the MNA collection while SEM pictures are available for ~31% of the species (some of which are already available on the Antarctic Field Guide project at: http://afg.biodiversity.aq/pdfs/144164-a-field-guide-to-antarctic-sponges.pdf).

**Figure 8.**
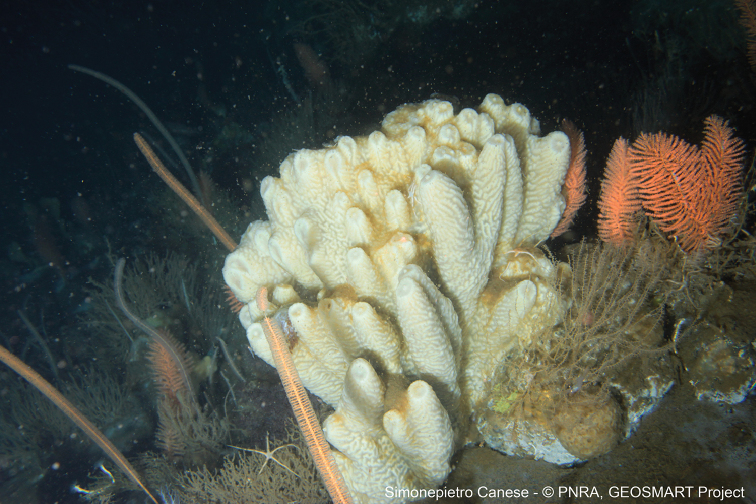
ROV video frame of the species Tedania (Tedaniopsis) oxeata Topsent, 1916 (MNA 8244) found at 250 meters of depth before the use of the ROV arm to obtain a fragment.

In a few cases (i.e. *Plakinamonolopha* Schulze, 1880, MNA 1715, MNA 1753; *Plakinatrilopha* Schulze, 1880, MNA 1504; *Euryponminiaceum* Thiele, 1905, MNA 9106), there is no voucher and only glass slides with spicules are available. Another species, i.e., Lycopodinacf.vaceleti (van Soest & Baker, 2011), is without a MNA collection code, as it was identified from an ROV video frame (Fig. 6), and represents the first record of this carnivorous sponge at Terra Nova Bay and, globally, the second record of this species (Van Soest and Baker 2011). This species was identified thanks to Rob Van Soest and Claire Goodwin and represents an important “visual record” because only a few pictures of carnivorous sponges *in situ* are available. In the case of the species Tedania (Tedaniopsis) oxeata Topsent, 1916 (MNA 8244), the specimen record was obtained by using the ROV arms (Fig. 8).

The MNA collection also includes several sponge holotypes (Table 1). Some species (i.e. *Haliclonissaverrucosa* Burton, 1932 with vouchers MNA 915, MNA 916, MNA 917, MNA 918; Anoxycalyx (Scolymastra) joubini (Topsent, 1916) with voucher MNA 928; Lissodendoryx (Ectyodoryx) nobilis (Ridley & Dendy, 1886) with voucher MNA 863) were previously published in other manuscripts (i.e. Fondi et al. 2014; Orlandini et al. 2014; Papaleo et al. 2013; 2012; Mangano et al. 2009; Romoli et al. 2011) without an MNA catalogue number which have been assigned after the publication. In the collection is also present an uncommon sponge (i.e. *Tethyopsisbrondstedi* (Burton, 1929), MNA 2839) which was previously sampled as a single specimen during the Terra Nova expedition in 1910, in the eastern sector of the Ross Sea, in particular in the regions of McMurdo Sound and Terra Nova Bay and at depth range of 402-965 meters (Burton 1929). According to records obtained from the Ocean Biogeographic Information System (OBIS 2018), this species had not been re-found for close to a century, until the NIWA (New Zealand National Institute of Water and Atmospheric Research) campaigns of 2004 and 2008 in the Ross Sea. During these recent expeditions, 17 specimens of this species were obtained. Two additional specimens of this small, idiosyncratic species have now been identified also from the 2002 MNA Carbonant expeditions in the Terra Nova Bay region. *Tethyopsis* Stewart, 1870, is a genus containing only nine known species, with the vast majority of these distinctive species occurring in the Southern Hemisphere, in particular, New Zealand and Antarctica (Van Soest et al. 2018). Online records of the global distributions of this genus from OBIS (2018) indicate that only ca. 200 specimens of this genus have been previously found prior to this publication.

**Table 1.** Sponge holotypes stored at the MNA.

MNA	Species
MNA 832	*Microxinasarai* Calcinai & Pansini, 2000
MNA 833	*Microxinalanceolata* Calcinai & Pansini, 2000
MNA 834	Lissodendoryx (Ectyodoryx) minuta Calcinai & Pansini, 2000
MNA 835	*Iophonterranovae* Calcinai & Pansini, 2000
MNA 865	Crella (Crella) aurantiaca Bertolino, Calcinai & Pansini, 2009
MNA 888	Mycale (Aegogropila) denticulata Bertolino, Calcinai & Pansini, 2009

### Taxonomic ranks

**Kingdom**: Animalia

**Phylum**: Porifera

**Class**: Calcarea

**Orders**: Clathrinida, Leucosolenida

**Families**: Achramorphidae, Grantiidae, Leucettidae, Sycettidae

**Genera**: *Leucetta*, *Leucandra*, *Megapogon*, *Sycetta*

**Species**: Leucandracf.gausapata (NR), Leucettidae sp.1 (NR), *Leucettaantarctica* (NR), *Megapogonraripilus* (NR), *Sycettaantarctica* (NR)

**Kingdom**: Animalia

**Phylum**: Porifera

**Class**: Demospongiae

**Orders**: Axinellida, Bubarida, Dendroceratida, Haplosclerida, Poecilosclerida, Polymastiida, Suberitida, Tetractinellida

**Families**: Acarnidae, Ancorinidae, Bubaridae, Chalinidae, Cladorhizidae, Coelosphaeridae, Crellidae, Darwinellidae, Dendoricellidae, Halichondriidae, Hymedesmiidae, Isodictyidae, Latrunculiidae, Microcionidae, Mycalidae, Myxillidae, Niphatidae, Phloedictyidae, Polymastiidae, Raspailiidae, Stylocordylidae, Suberitidae, Tedaniidae, Tetillidae

**Genera**: *Acanthorhabdus*, *Antarctotetilla*, *Artemisina*, *Asbestopluma*, *Bubaris*, *Calyx*, *Cinachyra*, *Clathria*, *Crella*, *Dendrilla*, *Eurypon*, *Fibulia*, *Halichondria*, *Haliclona*, *Haliclonissa*, *Hemigellius*, *Homaxinella*, *Hymeniacidon*, *Inflatella*, *Iophon*, *Isodictya*, *Kirkpatrickia*, *Latrunculia*, *Lissodendoryx*, *Lycopodina*, *Microxina*, *Mycale*, *Myxilla*, *Myxodoryx*, *Phorbas*, *Plicatellopsis*, *Plocamionida*, *Polymastia*, *Pseudosuberites*, *Sphaerotylus*, *Stylocordyla*, *Suberites*, *Tedania*, *Tethyopsis*

**Species**: *Acanthorhabdusfragilis* (NR), *Antarctotetillaleptoderma*, *Artemisinaapollinis* (NR), Artemisinacf.tubulosa, *Artemisina* sp.1 (NR), *Artemisinatubulosa*, Asbestopluma (Asbestopluma) belgicae, Asbestopluma (Asbestopluma) sp. (NR), Bubariscf.vermiculata (NR), *Bubarisvermiculata* (NR), *Calyxarcuarius*, *Calyxcf.arcuarius*, *Calyxcf.kerguelensis* (NR), *Cinachyraantarctica* (NR), *Cinachyrabarbata*, Cinachyracf.antarctica (NR), Cinachyracf.barbata, *Cinachyra* sp.1 (NR), Clathria (Axosuberites) nidificata, Clathria (Clathria) cf.toxipraedita, Clathria (Clathria) toxipraedita, Clathria (Microciona) antarctica (NR), Clathria (Thalysias) flabellata (NR), Crella (Crella) aurantiaca (NR), Crella (Pytheas) stylifera (NR), *Dendrillamembranosa*, *Euryponminiaceum*, *Fibuliacribriporosa* (NR), *Fibuliamaeandrina* (NR), Halichondria (Halichondria) panicea (NR), Haliclona (Gellius) cf.flagellifera (NR), Haliclona (Gellius) cf.glacialis (NR), Haliclona (Gellius) cf.spongiosa (NR), Haliclona (Gellius) glacialis (NR), Haliclona (Gellius) tylotoxa (NR), Haliclona (Haliclona) cf.penicillata, Haliclona (Haliclona) penicillata, Haliclona (Rhizoniera) cf.dancoi, Haliclonacf.divulgata (NR), Haliclonacf.scotti (NR), Haliclona (Rhizoniera) dancoi, *Haliclonadivulgata* (NR), Haliclona (Soestella) cf.chilensis (NR), *Haliclona* sp. (NR), *Haliclona* sp.1 (NR), *Haliclona* sp.2 (NR), *Haliclona* sp.3 (NR), *Haliclona* sp.4 (NR), *Haliclonavirens* (NR), *Haliclonissaverrucosa* (NR), *Hemigelliusbidens* (NR), *Hemigelliuscalyx* (NR), Hemigelliuscf.fimbriatus, *Hemigelliusfimbriatus*, *Hemigelliuspilosus*, *Homaxinellabalfourensis*, Homaxinellacf.balfourensis, Homaxinellacf.flagelliformis, *Homaxinellaflagelliformis*, *Hymeniacidoninsutus* (NR), *Inflatellabelli*, Inflatellacf.coelosphaeroides (NR), *Inflatella* sp.1 (NR), *Iophonterranovae*, *Iophonunicorne*, *Isodictyaconulosa*, *Isodictyaerinacea*, *Isodictyakerguelenensis*, *Isodictyamicrochela* (NR), *Isodictyasetifera*, *Isodictya* sp.1 (NR), *Isodictya* sp.2 (NR), *Isodictyatoxophila* (NR), *Kirkpatrickiacoulmani* (NR), *Kirkpatrickiavariolosa*, Latrunculia (Latrunculia) biformis, *Lissodendoryx* sp.1 (NR), Lissodendoryx (Ectyodoryx) anacantha (NR), Lissodendoryx (Ectyodoryx) antarctica, Lissodendoryx (Ectyodoryx) minuta, Lissodendoryx (Ectyodoryx) nobilis, Lissodendoryx (Ectyodoryx) ramilobosa, Lissodendoryx (Lissodendoryx) flabellata, Lycopodinacf.vaceleti (NR), *Microxinabenedeni*, Microxinacf.simplex (NR), *Microxinacharcoti*, *Microxinalanceolata*, *Microxinasarai*, *Microxinasimplex* (NR), *Microxina* sp.1 (NR), Mycale (Aegogropila) denticulata (NR), Mycale (Aegogropila) magellanica, Mycale (Mycale) tridens, Mycale (Oxymycale) acerata, *Mycalefibrosa*, *Mycale* sp.1 (NR), Myxilla (Burtonanchora) asigmata, Myxilla (Myxilla) elongata, Myxilla (Myxilla) mollis (NR), *Myxilla* sp.1 (NR), Myxodoryxcf.hanitschi, *Myxodoryxhanitschi*, *Phorbasglaberrimus*, *Phorbasnexus* (NR), *Plicatellopsisantarctica*, *Plocamionidagaussiana* (NR), *Polymastiainvaginata*, *Pseudosuberitesmontiniger*, *Pseudosuberitesnudus*, *Sphaerotylusantarcticus*, Stylocordylacf.chupachups, *Stylocordylachupachups*, *Suberitescaminatus*, *Suberitesmollis* (NR), *Suberites* sp.1 (NR), Tedania (Tedaniopsis) cf.charcoti, Tedania (Tedaniopsis) charcoti, Tedania (Tedaniopsis) massa (NR), Tedania (Tedaniopsis) oxeata (NR), Tedania (Tedaniopsis) tantula, *Tedania* sp.1 (NR), *Trachytedaniaspinata* (NR), *Tethyopsisbrondstedi* (NR)

**Kingdom**: Animalia

**Phylum**: Porifera

**Class**: Hexactinellida

**Order**: Lyssacinosida

**Family**: Rossellidae

**Genera**: *Anoxycalyx*, *Rossella*

**Species**: Anoxycalyx (Scolymastra) joubini, *Rossellaantarctica* (NR), Rossellacf.antarctica (NR), Rossellacf.longstaffi (NR), Rossellacf.villosa (NR), *Rossellafibulata* (NR), *Rossellalevis* (NR), *Rossellanuda* (NR), Rossellacf.nuda (NR), *Rossellaracovitzae* (NR), *Rossella* sp.1 (NR), *Rossella* sp.2 (NR), *Rossella* sp.3 (NR)

**Kingdom**: Animalia

**Phylum**: Porifera

**Class**: Homoscleromorpha

**Order**: Homosclerophorida

**Family**: Plakinidae

**Genus**: *Plakina*

**Species**: *Plakinamonolopha*, *Plakinatrilopha*

### Spatial coverage

**General geographic description**:

Ross Sea, Antarctica (Figs 1, 3, 4, 5)

**Coordinates**:

PNRA III expedition: -74.64833, -74.96667; 164.00000, 164.61167

PNRA V expedition: -74.63450, -74.90400; 164.02433, 164.47500

PNRA IX expedition: -74.71667, -75.76333; 164.04280, 164.19058

PNRA X expedition: -74.68557, -74.89367; 163.78557, 164.14752

PNRA XI expedition: -74.66833, -74.78333; 164.03333, 164.29167

PNRA XIII expedition: -74.71357, 164.13772

PNRA XIV expedition: -74.69405, -74.89950; 163.93748, 164.28620

PNRA XV expedition: -74.69667, -74.77658; 164.05327, 164.12868

PNRA XVII expedition: -72.51267, -76.76817; 164.09721, 179.50533

PNRA XVIII expedition: -77.56570, 163.61163

PNRA XIX expedition: -71.30667, -72.28667; 170.29833, 170.48667

PNRA XX expedition: -74.63347, -74.80570; 164.00194, 164.98583

PNRA XXI expedition: -74.69667, 164.08000

PNRA XXV expedition: -74.69027, -74.70348; 164.10255, 164.13762

PNRA XXVII expedition: -74.68562, -74.71337; 164.03502, 164.14903

PNRA XXVIII expedition: -74.68090, -74.77737; 163.95400, 164.23640

PNRA XXIX expedition: -74.68602, -74.72242; 164.03486, 164.24206

**Temporal coverage**:

PNRA III expedition: January 6, 1988 - February 2, 1988

PNRA V expedition: December 24, 1989 - February 1, 1990

PNRA IX expedition: December 27, 1993 - January 29, 1994

PNRA X expedition: January 21, 1995 - February 8, 1995

PNRA XI expedition: February 5, 1996 - February 8, 1996

PNRA XIII expedition: February 19, 1998

PNRA XIV expedition: January 6, 1999 - March 3, 1999

PNRA XV expedition: January 25, 2000 - April 25, 2000

PNRA XVII expedition: January 8, 2002 - February 7, 2002

PNRA XVIII expedition: November 11, 2002

PNRA XIX expedition: February 14, 2004 - February 16, 2004

PNRA XX expedition: January 17, 2005 - February 11, 2005

PNRA XXI expedition: January 23, 2006

PNRA XXV expedition: December 13, 2009 - January 11, 2010

PNRA XXVII expedition: January 10, 2012 - February 3, 2012

PNRA XXVIII expedition: January 9, 2013 - January 31, 2013

PNRA XXIX expedition: January 16, 2014 - February 1, 2014

### Natural collections description

**Parent collection identifier**: Italian National Antarctic Museum (MNA, section of Genoa, Italy)

**Collection name**: Porifera collection of the Italian National Antarctic Museum (MNA) - Data

**Specimen preservation method**: Part of the material collected during the expeditions was fixed in formalin and then transferred in ethanol (samples between 1985 and 2006), or was frozen immediately after collection and kept in the same condition in order to preserve the DNA quality and integrity. All samples are now stored in the collections of the Italian National Antarctic Museum (MNA, section of Genoa, Italy).

**Virtual collection of vouchers and 3D models**: The species used in the 3D model, *Tethyopsisbrondstedi* (Burton, 1929) (MNA 2839, Fig. 9) presents a spherical body from which 2 fragile oscular tubes protrude from the upper part of the main sponge body, reminiscent of a bull head with horns. The long oscular tubes are believed to serve as both inhalant and exhalant orifices for the sponge (Hajdu et al. 1994). The surface of the sponge’s main body is often found covered in small pebbles and other sandy debris, indicating that this species may live partially buried within the seabed sediment. The main sponge body is radial and is composed of oxeas (long spicules which are pointed at both ends) and triaenes (long spicules which are pointed at one end and the other is composed of three equal rays, reminiscent of a wind turbine). These triaenes can be bifurcated at each end of the ray (dichotriaene), the rays can be curved backwards (anatriaene), or are a triaene with only two rays, that are usually bifurcated at each end (orthodiaene). The oscular tubes of the sponge are composed of intricate layers of orthodiaenes. The main body of the sponge also contains tiny asters (star-shaped spicules, with rays radiating equally from a central point), which are composed of strongylasters (blunt-tipped or slightly bulbous ended rays) and oxyasters (with sharply pointed rays).

**Figure 9.** Video of the 3D model of *Tethyopsisbrondstedi* (Burton, 1929) (MNA 2839), a very uncommon sponge only occurring in the Ross Sea. The diameter of the main spherical body is ~10 mm and the length/width of the oscular tubes are respectively ~30 mm and ~5 mm.

The model of the sponge was obtained through micro-CT imaging performed at the Department of Geosciences (University of Padova) by CM. A bench-top Skyscan 1172 micro-CT system (Bruker®), equipped with a Hamamatsu 100/250 microfocus X-ray source (80 kV, 124 μA) and a Hamamatsu C9300 11 megapixel camera (with a pixel size of 8.68 μm) filtered by a 0.5 mm Aluminium foil was used. Projection images were acquired with 1200 ms exposure time, 2x2 binning mode, 0.30° rotation step over 360° rotation, averaged over 10 frames and in vertical random movement mode to minimise noise, providing an image pixel size of 13.2 μm. An oversized sample option was applied with 4 connected scans, leading to a total acquisition time of about 1170 min. Post-acquisition reconstruction was performed using the NRecon (Bruker microCT®) software package, starting from raw projection images, and applying thermal correction, misalignment compensation, ring artefact reduction and beam hardening correction. Segmentation was then performed with CT Analyser (Bruker microCT®) software package, using a 3D adaptive thresholding procedure (mean of minimum and maximum value) within spherical kernels of radius 8 pixels, starting from a pre-determined pre-thresholding value. Resulting images were saved as monochrome (1 bit) bitmaps and imported in the CTVox (Bruker microCT®) software package to perform 3D rendering and animations. The model will be available on the MNA web site (www.mna.it) and on Sketchfab (https://sketchfab.com/MNA). The species chosen for the model corresponded to one of the few specimens collected in the Ross Sea area after the species description (Burton 1929).

### Datasets

**Dataset description**: This dataset contains data about all four classes (Calcarea, Demospongiae, Hexactinellida and Homoscleromorpha) of the Phylum Porifera, based on vouchers from the Ross Sea (with a special focus on Terra Nova Bay) curated at the MNA. In total, the dataset includes 144 different morphospecies, and a total of 807 specimens. Several studies were based on this dataset: Alvizu et al. (in press); Bertolino et al. 2009; Calcinai et al. 2000; Cattaneo-Vietti et al. 2000; Fondi et al. 2014; Mangano et al. 2009; Orlandini et al. 2014; Papaleo et al. 2012, 2013; Romoli et al. 2011; Sarà 2002; Sarà et al. 1992. The validity and synonyms of each species name were checked in WORMS (World Register of Marine Species; http://www.marinespecies.org; last check made on 2018-03-28). The Darwin Core elements included in the dataset are: ID, Institution code (i.e. the name of the institution where the samples are kept), basis of record, occurrence ID, catalogue number (i.e. MNA catalogue number), individual count, preparation (preservation method and more info about the sample, e.g., ETOH, dry, glass slides, etc.), event ID (i.e. original sampling station code), sampling protocol (sampling gear), event date, year, month, day, verbatim event date, field number (sampling station code as showed in the maps), event remarks (i.e., expedition), maximum depth meters, decimal latitude, decimal longitude, taxon ID, scientific name ID, scientific name, kingdom, phylum, class, order, family, genus, subgenus, specificEpithet, infraspecificEpithet, scientific name authorship, and taxon remarks. Some of the sampling stations are dredge stations, which have two sets of coordinates: the starting and end points. In these cases the coordinates reported in the dataset refer to the starting point of the dredge station.

**Object name**: Porifera collection of the Italian National Antarctic Museum (MNA) - Data

**Character encoding**: UTF-8

**Format name**: Darwin Core Archive format

**Format version**: 1.0

**Distribution**: http://ipt.biodiversity.aq/resource.do?r=mna_antarctic_porifera

**Language**: English

**Metadata language**: English

**License of use**: This dataset [Porifera collection of the Italian National Antarctic Museum (MNA) - Data] is made available under the Creative Commons Attribution License (CC-BY) 4.0: http://www.creativecommons.org/licenses/by/4.0/legalcode

**Date of metadata creation**: 2018-03-28

**Hierarchy level**: Dataset
